# Dabigatran Added to Dual Antiplatelet Therapy to Treat a Left Ventricular Thrombus in an 87 Year Old Patient With Myocardial Infarction and Very High Bleeding Risk

**DOI:** 10.3389/fphar.2018.00217

**Published:** 2018-04-04

**Authors:** Maria Noflatscher, Nicolas Moes, Eva-Maria Gassner, Peter Marschang

**Affiliations:** ^1^Angiology, Department of Internal Medicine III Cardiology, Innsbruck Medical University, Innsbruck, Austria; ^2^Department of Radiology, Innsbruck Medical University, Innsbruck, Austria

**Keywords:** triple therapy, left ventricular thrombus, myocardial infarction, high bleeding risk, anticoagulation therapy, rectal neoplasms

## Abstract

**Background:** A left ventricular (LV) thrombus is detected in approximately 5–10% of patients after myocardial infarction (MI). If left untreated, these LV thrombi carry a significant risk of complications including embolic stroke. According to current guidelines, anticoagulation with vitamin K antagonists (VKA) is recommended to treat a LV thrombus.

**Case presentation:** An 87 year old patient was referred to our department with non ST-elevation MI. Five months before, he had been diagnosed with a subacute ST elevation MI, which had been treated conservatively. Recently, a rectal neoplasia had been diagnosed, but not operated yet. The patient underwent coronary angiography with implantation of two drug eluting stents (Cre8) requiring dual antiplatelet therapy. During ventriculography an apical LV thrombus of 16 mm diameter was detected. Due to the high bleeding risk in this patient, VKA therapy with potentially fluctuating international normalized ratio (INR) values was considered unsuitable. Therefore, dabigatran at a dose of 110 mg bid was chosen as anticoagulation therapy. After 4 weeks, cardiac computed tomography was performed, which failed to detect the LV thrombus described previously. Notably, triple therapy with dabigatran, clopidogrel, and aspirin was well tolerated without evidence for bleeding. The surgical resection of the rectal neoplasm was performed 2 months later without bleeding complications.

**Discussion:** Anticoagulation is effective in patients with MI and a LV thrombus in reducing the risk of embolization and in dissolving the thrombus. Our case is complex due to the required triple therapy, very old age and significant bleeding risk of our patient due to the rectal neoplasia. Although only few reports are available for the use of non VKA oral anticoagulants (NOAC) in this indication, we chose dabigatran at a dose of 110 mg bid added to dual antiplatelet therapy for our patient. Besides the advantage of a predictable pharmacokinetic profile of NOAC in contrast to VKA, the effect of dabigatran can rapidly be reversed by idaruzicumab in the case of severe bleeding.

**Conclusion remarks:** Physicians should carefully weigh the risk of thromboembolic events versus the risk of bleeding when combining antiplatelet with anticoagulation therapy.

## Introduction

An 87 year old patient was referred to our department with a non-ST-elevation myocardial infarction (MI). Five months before, he had been diagnosed with a subacute ST elevation MI, which had been treated conservatively. His medical history included a transient ischemic attack with subsequent endarterectomy in 2007, an infrarenal aortic aneurysm (max diameter 3, 6 cm) as well as arterial hypertension and diabetes mellitus type II. Shortly before this event, copidogrel, and aspirin had been interrupted due to a planned operation of a newly diagnosed rectal carcinoma.

## Background

The formation of a left ventricular (LV) thrombus is not an unusual finding in patients with MI. In the literature, different frequencies of LV thrombi after MI have been reported. Solheim et al. described a frequency of 15% in the first 3 months after anterior wall MI (Solheim et al., 2010). Zielinska et al. demonstrated an incidence of 2.5% in patients with an acute MI (Zielinska et al., 2008). Recent reviews state the incidence of LV thrombus formation after ST-elevation MI to be approximately 5–10%. Due to more aggressive anticoagulation therapies in the acute phase and improved LV remodeling, the incidence of LV thrombi appears to be declining (Delewi et al., 2012). Presently, only a few case reports are available on the use of new oral anticoagulants (NOAC) in the literature.

## Diagnosis

**Figure 1:**
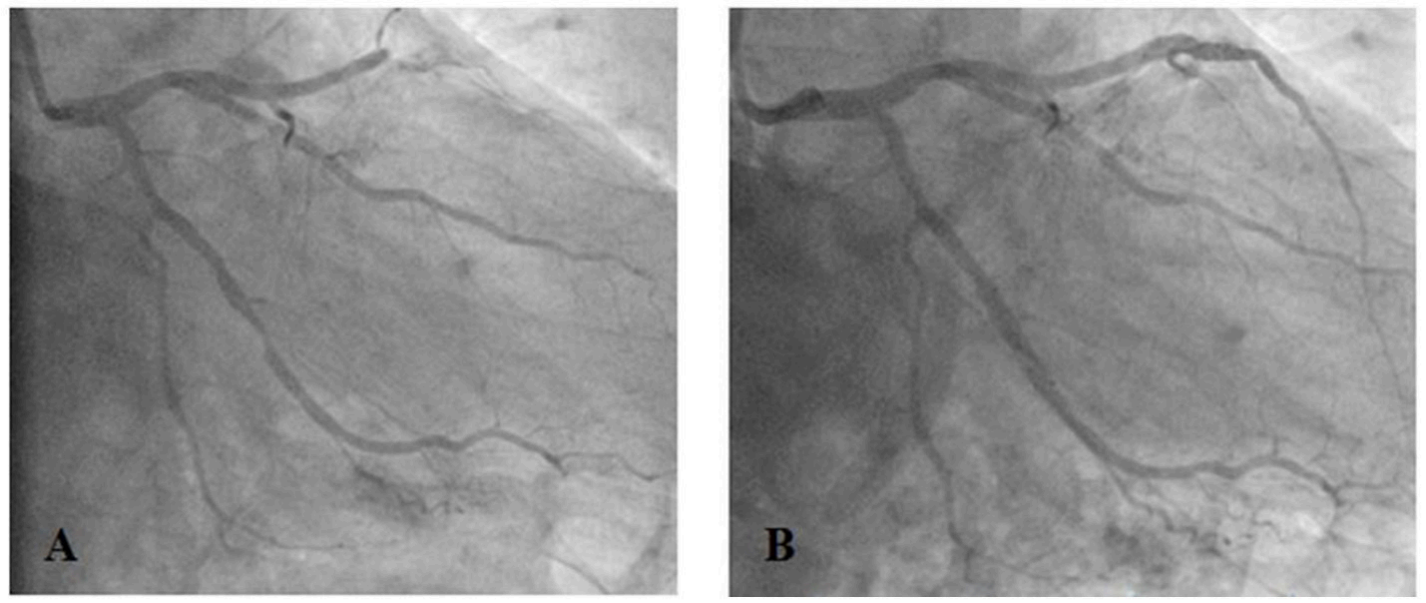
Proximal occlusion of the left anterior descending artery (LAD) and significant (80%) stenosis of the circumflex artery (CX) **(A)** treated by implantation of drug eluting stents (Cre8 3 × 25 mm into the LAD, Cre8 3.6 × 16 mm into the CX) **(B)**.

Due to the diagnosed non-ST-elevation MI coronary angiography was performed. The exam showed a proximal occlusion of the left anterior descending (LAD) artery and significant (80%) stenosis of the circumflex (CX) artery (Farooq et al., 2012), which were treated by implantation of two polymer-free drug eluting stents (DES) (Cre8 3 × 25 mm into the LAD, Cre8 3.6 × 16 mm into the CX) (Figure 1).

**Figure 2:**
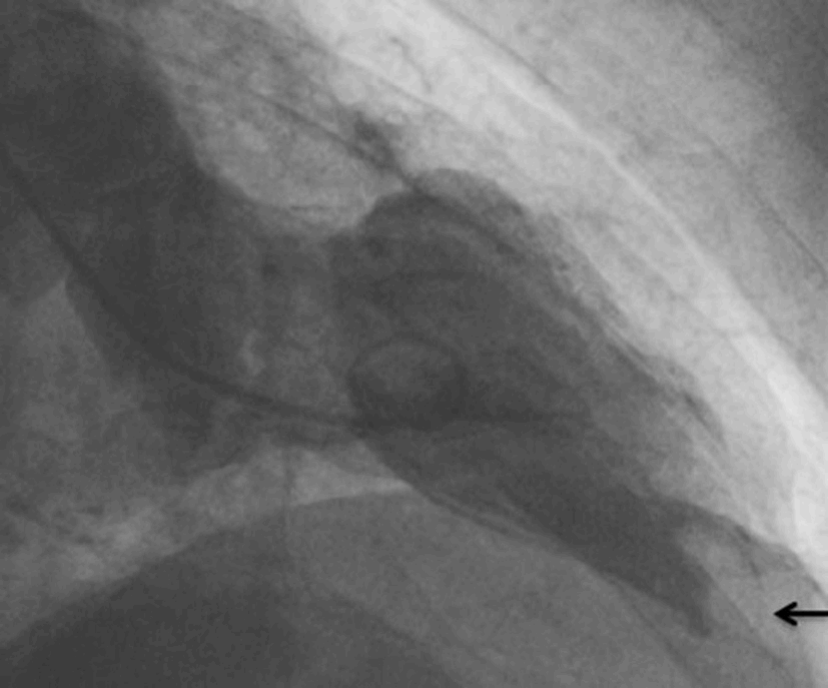
Thrombus (arrow) in the apical part of the left ventricle as demonstrated by ventriculography.

Ventriculography showed a moderately reduced global LV function (ejection fraction of 40%) with apical and septal akinesia. In addition, a thrombus (16 mm diameter) was clearly visualized in the apical part of the left ventricle (Figure 2).

## Treatment

Due to the acute coronary syndrome (ACS) with implantation of two DES, dual antiplatelet therapy (clopidogrel 75 mg and aspirin 100 mg) was started. In addition, oral anticoagulation therapy had to be initiated to treat the detected thrombus in the left ventricle. Because of the high bleeding risk in this patient with a newly diagnosed, not yet operated rectal neoplasia, vitamin K antagonist (VKA) therapy with potentially fluctuating international normalized ratio (INR) values was considered unsuitable. Therefore, dabigatran was chosen as anticoagulation therapy. Since the patient showed slightly reduced renal function parameters (creatinine clearance 58 ml/min/1.73 m2, creatinine 1.18 mg/dl) the reduced dose of dabigatran (of 110 mg bid) was used.

**Figure 3:**
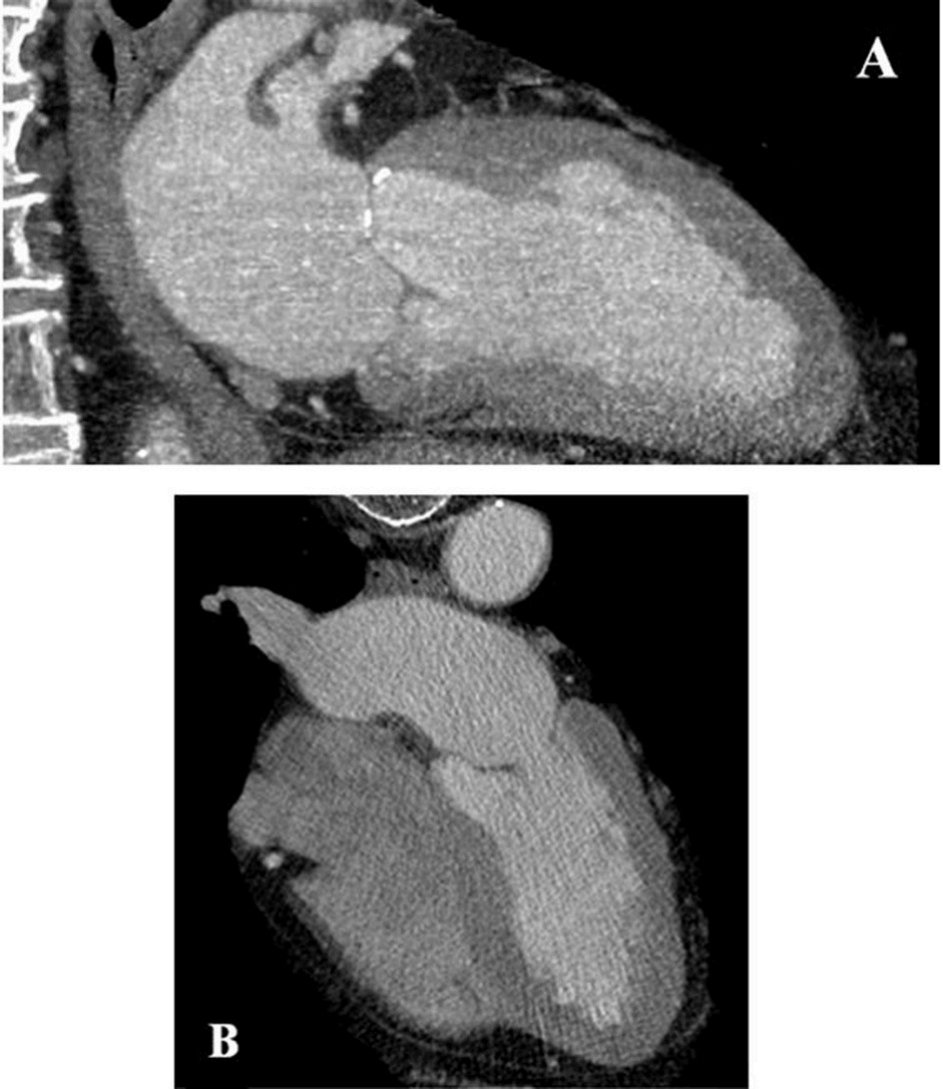
Cardiac computed tomography of the left ventricle vertical view **(A)** and four chamber view **(B)** without any evidence for a left ventricular thrombus after 4 weeks.

After 4 weeks, the patient was seen again in our outpatient clinic. Notably, triple therapy with dabigatran, clopidogrel, and aspirin had been well tolerated without evidence for significant bleeding. At this time, a cardiac computed tomography was performed, which failed to detect the LV thrombus described previously (Figure 3). Consequently, dabigatran therapy was stopped and dual antiplatelet therapy was continued for another 2 months. The patient was then switched to aspirin monotherapy and the surgical resection of the rectal neoplasm was performed in a peripheral hospital without any bleeding complications and stable hemoglobin values during and after the surgical resection.

## Discussion

If left untreated, a LV thrombus may lead to devastating complications, most notably embolic stroke. The risk of embolization in patients who are not treated with anticoagulant therapy is between 10 and 15%. Most embolic events occur within the first 3–4 months (Stratton and Resnick, 1987). Patients with MI and LV thrombus treated with anticoagulation therapy have a better outcome due to the reduced risk of embolic events (Vaitkus and Barnathan, 1993).

Due to the lack of data from clinical studies for NOAC in the treatment of LV thrombi, VKA therapy is usually initiated in these patients. VKA have been used for atrial and ventricular thrombi (Nagamoto et al., 2014). Nieman et al. reported that anticoagulation with warfarin therapy can resolve fresh thrombi, but not chronic ventricular thrombi after MI (Niemann et al., 2012). However, several case reports have shown the feasibility of NOAC treatment of LV thrombi with dabigatran (Kaku, 2013; Nagamoto et al., 2014; Chung et al., 2015; Kolekar et al., 2015; Ohashi et al., 2015) or factor Xa inhibitors (Nakasuka et al., 2014; Padilla Pérez et al., 2014; Kaya et al., 2016; Makrides, 2016; Mano et al., 2016; Berry et al., 2017; Seecheran et al., 2017; Smetana et al., 2017). In the cases describing dabigatran to resolve LV thrombi, the doses varied between 110 and 150 mg bid and the resolution of the thrombi was confirmed after 3 weeks to 4 months (Hori et al., 2013).

The European Society of Cardiology—(ESC) Guidelines of 2014 recommend the use of new-generation DES over BMS (bare metal stents) (European Heart Rhythm et al., 2010), because of their lower rate of restenosis-and stent thrombosis (Lip et al., 2014). The risk for stent thrombosis is highest in the first months after stent implantation and dual antiplatelet therapy is recommended for at least 4 weeks (Levine et al., 2011).

Our case is particularly complex and unusual because of the required triple therapy, very old age and significant bleeding risk of our patient due to the newly diagnosed rectal carcinoma. Therefore, we considered VKA therapy for this patient with potentially fluctuating INR values unsuitable. We chose dabigatran due to the high bleeding risk of our patient and the availability of an efficient antidote (idarucizumab), which had shown rapid and complete reversion of the anticoagulant activity of dabigatran in the RE-VERSE AD study (Pollack et al., 2015). Only few cases have been reported in the literature where triple therapy was used to resolve a LV thrombus with Factor Xa inhibitors (Makrides, 2016; Mano et al., 2016; Berry et al., 2017; Seecheran et al., 2017; Smetana et al., 2017) or dabigatran (Chung et al., 2015; Ohashi et al., 2015). In contrast to these described cases, apparent development of a LV thrombus under dabigatran treatment has been published recently (Adar et al., 2018).

For patients requiring NOAC therapy in combination with clopidogrel and/ or low-dose aspirin, the ESC-Guidelines recommend the use of the lower dose of the NOAC, in the case of dabigatran 110 bid (Connolly et al., 2009). Therefore, and due to the considerable bleeding risk, we used dabigatran in the lower dose (110 mg bid) for our patient.

However, similar as VKA, combination of dabigatran with dual antiplatelet therapy carries an increased risk of major bleeding (Dans et al., 2013; Oldgren et al., 2013).

The RE-DEEM study showed a dose depending bleeding risk for dabigatran in combination with dual antiplatelet therapy. The addition of dabigatran to dual antiplatelet therapy in patients with recent ACS showed a reduction in major adverse cardiovascular events but an essential increase in bleeding compared to dual and single antiplatelet therapy alone (Oldgren et al., 2011).

The recently published RE-DUAL-PCI-Study represents an additional option in patients with risk of bleeding and thromboembolic events. This study showed a lower risk of bleeding and equal risk of thromboembolic events using dual therapy in patients with atrial fibrillation after percutaneous coronary intervention (PCI) with dabigatran (110 or 150 mg twice daily) and a P2Y12 inhibitor (clopidogrel or ticagrelor) compared to triple therapy with warfarin plus a P2Y12 inhibitor (clopidogrel or ticagrelor) and aspirin (for 1–3 months) (Cannon et al., 2017).

## Concluding remarks

Physician should carefully weigh the risk of thromboembolic events versus the risk of bleeding when combining antiplatelet with antithrombotic therapy.

## Ethics statement

The study is a case report describing routine treatment of a single patient and is therefore exempt from ethical approval procedures.

## Author contributions

MN: Drafting the work, analysis and interpretation of data for the work, final approval of the version to be published, agreement to be accountable for all aspects of the work. NM: Performing the coronary angiography of the patient, final approval of the version to be published, agreement to be accountable for all aspects of the work, revising the work critically for important intellectual content. E-MG: Analysis and interpretation of the CT-Images, final approval of the version to be published, agreement to be accountable for all aspects of the work, revising the work critically for important intellectual content. PM: Analysis and interpretation of data for the work, final approval of the version to be published, agreement to be accountable for all aspects of the work in ensuring that questions related to the accuracy or integrity of any part of the work, revising the work critically for important intellectual content.

### Conflict of interest statement

The authors declare that the research was conducted in the absence of any commercial or financial relationships that could be construed as a potential conflict of interest.

## Abbreviations

bid: bis in die, twice a day
BMS: bare metal stent
cm: centimeters
CX: circumflex artery
DES: drug eluting stent
Dl: decilitres
ESC: European Society of Cardiology
et al.: and others
Fig: figure
INR: international normalized ratio
LAD: left anterior descending
LV: left ventricular
m2: square meters
mm: millimeters
mg: milligrams
MI: myocardial infarction
min: minutes
ml: milliliters
NOAC: new oral anticoagulants
VKA: vitamin K antagonist
PCI: percutaneous coronary intervention.
